# Gender differences among professionals dedicated to Oral Implantology in Spain: An observational study

**DOI:** 10.4317/jced.59263

**Published:** 2022-02-01

**Authors:** Angel-Orión Salgado-Peralvo, Juan-Francisco Peña-Cardelles, Naresh Kewalramani, Alvaro Jiménez-Guerra, Eugenio Velasco-Ortega, Loreto Monsalve-Guil

**Affiliations:** 1 DDS, MSc, MSc, PhD. Department of Stomatology, Faculty of Dentistry, University of Seville, Seville, Spain; 2 DDS, MSc, PhD. Department of Basic Health Sciences, Rey Juan Carlos University, Madrid, Spain; 3 DDS, MSc. Department of Nursery and Stomatology, Rey Juan Carlos University, Madrid, Spain; 4 MD, PhD. Department of Stomatology, Faculty of Dentistry, University of Seville, Seville, Spain

## Abstract

**Background:**

Despite the development of society and the educational progress achieved at the university education level, women continue to face obstacles that hinder their professional development. This study aims to determine whether there are gender differences in a representative sample of professionals dedicated to Oral Implantology in Spain.

**Material and Methods:**

This is a cross-sectional observational study based on the STROBE (Strengthening the Reporting of Observational Studies in Epidemiology) guidelines. An electronic survey consisting of two blocks of questions was sent to members of the Spanish Society of Implants. The data were analyzed using descriptive analysis.

**Results:**

A total of 303 participants (20.8%) responded to the questionnaire, of which 219 were men (72.3%) and 84 women (27.7%). Up to the age of 40 years, women predominate, whereas men predominate from the age of 51 years onwards, which is influenced by a greater number of years of experience in implant placement and a higher number of implants placed per year. Despite this, women have a higher level of training in Oral Implantology, as a greater proportion are trained through master’s degrees.

**Conclusions:**

The greater representation of men in the study is associated with the ageing of the sample. The results obtained from the present study anticipate the trend of a greater presence of the female gender in Oral Implantology in Spain in the coming years.

** Key words:**Sex differences, dentistry, oral implantology, feminism, gender.

## Introduction

In recent years, there has been a significant increase in the number of women in the health sciences undergraduate degrees and, by extension, in dentistry (1,2). Despite this, women often encounter challenging attitudes and/or barriers to continuing or advancing in their profession. These include reasons such as lack of female mentors and role models, a greater burden of responsibility for parenting, lack of parity in rewards such as career advancement and salary, gender discrimination and sexual harassment (3).

The influences of biological sex and gender, as well as the interactions between them, need to be explored to understand the challenges women dentists face in their careers. “Sex” is a biological concept and “gender” is a social construct that specifies the roles that men and women must follow socially and culturally (4). In this sense, family care responsibilities continue to fall disproportionately on women. This may explain why women abandon their careers at an advanced stage.

Thus, although the profile of the dental profession has changed, career paths in dentistry, in many cases, continue to be gender-biased. A distinction can be made between horizontal and vertical gender segregation. Horizontal segregation is evident from differences in the choice of speciality, with female dentists preferring paediatric dentistry or orthodontics, and less interested in others, such as oral surgery, which are more associated with the male gender (5) In contrast, vertical gender segregation is reflected in the dental hierarchy. The highest positions in the academic field are mainly occupied by male dentists (6).

On the other hand, a recent literature review showed that there are usually fewer female dental centre owners, they work 4-6 h less per week and see fewer patients than men, which ultimately translates into a wage gap. Also, there tends to be a lower proportion of women specialists, in university positions and leadership roles (7).

This study aims to determine whether there are gender differences in a representative sample of professionals dedicated to Oral Implantology in Spain.

## Material and Methods

- Study design

A cross-sectional observational study was carried out following STROBE (8) (Strengthening the Reporting of Observational Studies in Epidemiology) guidelines. Prior to the study, approval was obtained from the Ethics Committee of the Spanish Society of Implants (SEI – Sociedad Española de Implantes).

- Questionnaire

A questionnaire was sent via Google Drive and was open to respondents from April to July 2020, during which time two reminders were sent so that those who had not answered the questionnaire could do so. The survey consisted of two blocks of closed questions. The first of these, consisting of 8 closed questions, inquired about general data relating to the professionals surveyed, such as demographic data, data related to academic training in the field of Oral Implantology, Oral Surgery, Periodontology, and/or their combinations, and professional data. Finally, a second block inquired about the treatments related to the implant placement.

- Participant Recruitment

The survey was sent to all SEI members who did not express their wish not to receive e-mails (n= 1,460). Completion of the survey implied the participant’s consent to the collection of this information. The final sample size consisted of those professionals who chose to fully respond to the survey (n= 303). Each participant could only respond to the electronic survey once, and the options for each question, as well as the questionnaire variables, are shown in Tables  Table 1 and  Table 2. There could be no selection bias, as the electronic survey was sent to all dentists registered in the SEI.

**Table 1:**
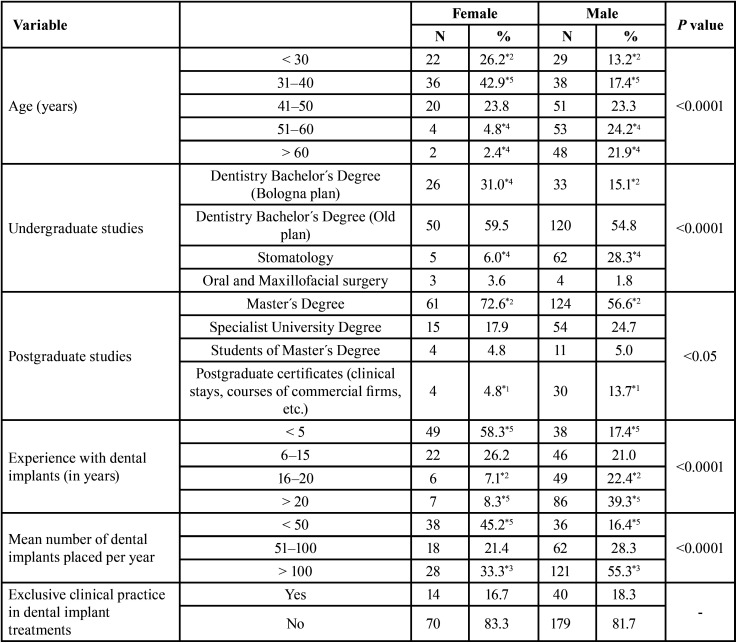
Demographic and professional characteristics of the study sample (Statistical significance of the cell: *1: *p*<0.05; *2: *p*<0.01; *3: *p*<0.001, *4: *p*<0.0001 y *5: *p*<0.00001).

**Table 2:**
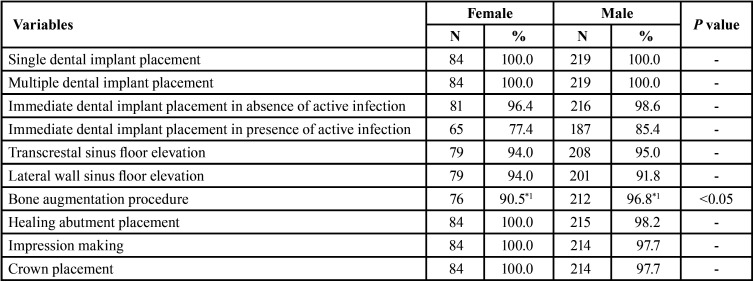
Treatments performed according to the gender of the professional (Statistical significance of the cell: *1: *p*<0.05).

- Statistical analysis

Questions were entered into Google Forms for electronic distribution and data collection. Data was exported to Microsoft Excel for cleaning and manipulation of variables for further analysis. The collected data were analyzed using IBM® SPSS Statistics v.26 (IBM® Corp., Armonk, NY, USA); 95% confidence intervals (CI) were applied. All study variables were treated quantitatively. A normality test was previously applied, observing that no variable followed a normal distribution, so the Mann–Whitney U test was applied for the crossover for dichotomous variables and Kruskal–Wallis for variables with more than two categories. The chi-squared test was used.

## Results

- Demographics

A total of 303 responses were obtained, representing a response rate of 20.8%. Being one of the three scientific societies currently existing in Spain related to implant placement, the study sample is considered representative. All the participants answered all the questions in the survey, as all the questions were established as compulsory, i.e., if a question was not answered, it was not possible to move on to the next one.

- Main data

Of the 303 respondents, 219 were male (72.3%) and 84 were female (27.7%). Concerning age, significant differences were found between men and women (*p*<0.0001), with a higher proportion of women (69.1%) than men (30.6%) up to the age of 40. Specifically, up to the age of 30, 22 women (26.2%; *p*<0.01) participated in the survey compared to 29 men (13.2%; *p*<0.01) and from the age of 31 to 40, 36 women (42.9%; *p*<0.00001) compared to 38 men (17.4%; *p*<0.00001). From the age of 51 onwards, the opposite is observed, with men (46.1%) predominating over women (7.2%). More specifically, from 51 to 60 years of age, 4 women participated (4.8%; *p*<0.001) compared to 53 men (24.2%; *p*<0.001) and two women over 60 years of age (2.4%; *p*<0.001) compared to 48 men (21.9%; *p*<0.001).

Differences were also observed in terms of undergraduate education (*p*<0.0001), with a higher proportion of women being dental graduates (n= 26; 31.0%; *p*<0.001) compared to men (n= 33; 15.1%; *p*<0.01), while the majority of stomatologists who responded to the survey were men, with 62 (28.3%; *p*<0.0001) responding compared to 5 women (6.0%; *p*<0.0001). These differences were also found in postgraduate education (*p*<0.05), with a lower proportion of men (n=124; 56.6%; *p*<0.01) having completed a master’s degree compared to women (n= 61; 72.6%; *p*<0.01). The male gender was also more represented in the completion of training courses, with 30 males (13.7%; *p*<0.05) compared to 4 females (4.8%; *p*<0.05).

Similarly, gender differences in experience were observed, manifested as the number of years performing implant treatments and the mean number of implants placed per year (*p*<0.0001). Concerning the first of these, the women surveyed have been placing implants for fewer years. In this regard, 49 women (58.3%; *p*<0.00001) have experience of up to 5 years compared to 38 men (17.4%; *p*<0.00001). In contrast, 6 women (7.1%; *p*<0.01) have 16 to 20 years of experience compared to 49 men (22.4%; *p*<0.01), and 7 women (8.3%; *p*<0.00001) compared to 86 men (39.3%; *p*<0.00001) with more than 20 years of experience. On the other hand, men placed more implants per year than women. Specifically, 38 women (45.2%; *p* <0.00001) placed up to 50 implants per year compared to 36 men (16.4%; *p*<0.00001), and 28 women (33.3%; *p*<0.001) placed more than 100 implants compared to 121 men (55.3%; *p*<0.001).

Regarding the treatments performed, these were very similar in both men and women, except for bone augmentation procedures (*p*<0.05), with 212 men (96.8%; *p*<0.05) performing them compared to 76 women (90.5%; *p*<0.05) (Table 2).

## Discussion

The number of men surveyed was 44.6% higher than that of women (72.3% vs. 27.7%, respectively). Women’s access to Spanish universities was regulated in 1910. Since then, their presence has gradually increased. In this sense, in the studies of Medicine, the female gender went from 5.5% in the academic year 1940/41 to 20.2% in the academic year 1969/70 (9) In the last quarter of the 20th century, coinciding with the democratic period, there was a substantial change for women at the university level (10). Data published by the Spanish Ministry of Education (11) show that, during the 2008/09 academic year, the percentage of women enrolled in Spanish universities was 54.4% compared to 45.6% of men. In the 2017/18 academic year and, for the undergraduate level of education, the rate of female enrolment was 52.3% compared to 33.8% of male enrolment. In the 2019/20 academic year, the representation of women in the Health Sciences field was 70.8% (1). These data are in line with those obtained in this study, observing a clear female predominance in ages younger than 41 years, matching the male gender in the age category from 41 to 50 years, and a noTable increase of men from 51 years onwards, the latter age range representing 59.74% of the sample (n= 181). An additional reason for the lower proportion of women in our study could be a lower preference for surgical specialities (including Oral Implantology) (12,13), perhaps because, in general, men place more value on financial rewards and manual dexterity skills when choosing their “speciality”, while women’s motivation is focused on personal rewards (14,15), with specialities such as Orthodontics and/or Paediatric Dentistry being more attractive.

Gender differences in age and undergraduate education are related to the fact that, until 1987, the training of dentists in Spain was a medical speciality. In that year, a study plan for Dentistry was inaugurated, independent of Medicine, and later, in 2009, it was brought into line with the existing study plan in Europe, known as the Bologna Plan. In terms of postgraduate training, more women than men choose to specialise in implant treatment through master´s degrees (72.6% vs. 56.6%; *p*<0.01), while a higher proportion of men have taken non-accredited training courses than women (4.8% vs. 13.7%; *p*<0.05). This may be related to the presence of older men and the relatively recent development of master´s degrees.

On the other hand, the men in the sample have more experience in implant placement than the women in terms of the average number of years performing these treatments, as well as a higher number of implants placed per year, related to the age distribution. In other words, there is a significantly higher number of men over 51 years of age, which is associated with a higher number of years performing implant treatments (> 20 years) compared to women (39.3% vs. 8.3%; *p*<0.00001), while the percentage of women with experience of up to 5 years is significantly higher than that of men (58.3% vs. 17.4%; *p*<0.00001). The same results can be extrapolated to the mean number of implants placed per year, with the female gender predominating over the male gender in the placement of up to 50 implants per year (45.2% vs. 16.4%; *p*<0.00001), reversing when analysing the placement of more than 100 implants/year (33.3% vs. 55.3%, respectively; *p*<0.001). This greater experience is not reflected in a smaller range of treatments related to implant placement, except for bone augmentation procedures, where a significantly greater number of men perform these treatments compared to women (*p*<0.05).

Even though male representation is significantly higher in the sample studied, it is foreseeable that in approximately 15 years there will be a clear feminisation of Oral Implantology in Spain, coinciding with the retirement of professionals who are currently over 51 years of age. Feminisation can be defined as (a) an increase in female representation in a given profession and/or (b) changes in the current practices of a profession given the participation of women (16). This feminisation is not only manifested in the subgroup of oral implantologists but also the profession in general. In this regard, the General Council of Dentists of Spain observed an increase in the number of women from 1994 (29.54%) to 2020 (44.37%) (2).

- Limitations and future lines of research

As this is a survey-based study, it is not possible to establish with certainly the veracity of the answers provided by the participants. Future lines of research should be aimed at finding out whether there are differences in salaries and on the possibility of being able to combine personal and family life with work, to identify barriers that could equal professional development for men and women.

## Conclusions

The gender differences found in this study are related to the latter access of women to the university environment. However, the greater representation of men in the sample studied is associated with a greater presence of older respondents. This fact means that the male respondents have more experience in years of practice with a lower level of postgraduate training compared to the female gender. The results obtained from the present study anticipate the trend of a greater presence of the female gender in Oral Implantology in Spain.

## References

[1] (2020). Estadística de estudiantes.

[2] (2021). La demografía de los dentistas en España. Situación pasada, presente y futura: Análisis 1994-2020.

[3] Pinn VW (2006). Women's health research and health leadership: Benchmarks of the continuum. J Dent Educ.

[4] Risberg G, Johansson EE, Westman G, Hamberg K (2003). Gender in medicine - an issue for women only? A survey of physician teachers' gender attitudes. Int J Equity Health.

[5] Scarbecz M, Ross JA (2007). The relationship between gender and postgraduate aspirations among first- and fourth-year students at public dental schools: a longitudinal analysis. J Dent Educ.

[6] Pallavi SK, Rajkumar GC (2011). Professional practice among woman dentist. J Int Soc Prev Community Dent.

[7] McKay JC, Quiñonez CR (2012). The feminization of dentistry: implications for the profession. J Can Dent Assoc.

[8] von Elm E, Altman DG, Egger M, Pocock SJ, Gøtzsche PC, Vandenbroucke JP (2008). The Strengthening the Reporting of Observational Studies in Epidemiology (STROBE) statement: guidelines for reporting observational studies. J Clin Epidemiol.

[9] Santesmases Navarro de Palencia MJ (2000). Mujeres científicas en España (1940-1979). Profesionalización y modernización social.

[10] López-Cruz L (2002). La presencia de la Mujer en la universidad española. Rev Hist La Educ Latinoam.

[11] (2010). Estadística alumnado universitario. Avance de la estadística de estudiantes universitarios 2008-2009.

[12] Navarro-Mora M, Cartes-Velásquez R (2015). Expectativas de especialización profesional en estudiantes de Odontología. Revisión de la literatura. Rev Estomatológica Hered.

[13] Buddeberg-Fischer B, Klaghofer R, Abel T, Buddeberg C (2006). Swiss residents' speciality choices--impact of gender, personality traits, career motivation and life goals. BMC Health Serv Res.

[14] Zarchy M, Kinnunen T, Chang BM, Wright RF (2011). Increasing predoctoral dental students' motivations to specialize in prosthodontics. J Dent Educ.

[15] Lambert EM, Holmboe ES (2005). The relationship between specialty choice and gender of U.S. medical students, 1990-2003. Acad Med.

[16] Muzzin LJ, Brown GP, Hornosty RW (1994). Consequences of feminization of a profession: the case of Canadian pharmacy. Women Health.

